# The Transcription Factor StuA Regulates Oxidative Stress-Responsive Genes in *Trichophyton rubrum*

**DOI:** 10.3390/ijms252312959

**Published:** 2024-12-02

**Authors:** Monise Fazolin Petrucelli, Leonardo Martins-Santana, Vanderci M. Oliveira, Pablo R. Sanches, Antonio Rossi, Nilce M. Martinez-Rossi

**Affiliations:** Department of Genetics, Ribeirão Preto Medical School, University of São Paulo, Ribeirão Preto 14049-900, SP, Brazil; monisepetrucelli@usp.br (M.F.P.); leonardo.lms95@gmail.com (L.M.-S.); cuca@fmrp.usp.br (V.M.O.); psanches@usp.br (P.R.S.); anrossi@usp.br (A.R.)

**Keywords:** oxidative stress, transcription factors, glutathione, catalase, *Trichophyton rubrum*, StuA, HOG pathway, AP1-SKN7, hydrogen peroxide

## Abstract

Fungi can remarkably sense and adapt to various extracellular stimuli and stress conditions. Oxidative stress, which results from an imbalance between reactive oxygen species production and antioxidant defenses, leads to cellular damage and death. In *Trichophyton rubrum*, oxidative stress is managed by a complex antioxidant system, including thioredoxins, glutathione, catalases, peroxidases, and superoxide dismutase, with glutathione playing a crucial role. The fungus also responds to oxidative stress through critical pathways such as the glycerol high-osmolarity pathway, activator protein 1 transcription factor, and responsive regulator SKN7. To better understand the role of the transcription factor StuA in regulating oxidative stress-related genes within these pathways, we conducted gene expression studies in Δ*stuA* mutant and wild-type strains of *T. rubrum* cultivated in keratin and under oxidative stress induced by hydrogen peroxide. Our results revealed significant downregulation of essential antioxidant genes, including glutathione transferases and catalases, in the Δ*stuA* mutant. Moreover, catalase and glutathione S-transferase activities were impaired in the mutants under stress conditions, highlighting the impact of this mutation. These findings underscore the critical role of StuA in the oxidative stress response and fungal pathogenesis and provide new insights into *T. rubrum*’s adaptive mechanisms.

## 1. Introduction

*Trichophyton rubrum* is the most common agent of dermatophytosis worldwide, primarily causing cutaneous infections in keratin-rich structures, such as nails and skin. This fungus secretes proteolytic enzymes that degrade keratinized structures, which are metabolized and used as a nutritional source during infection [1,2]. *T. rubrum* senses and adapts to the host environment to survive and thrive during infection, responding appropriately to environmental cues and the cellular stresses they induce. To this end, the fungus has evolved diverse adaptation mechanisms, including a complex interplay between signaling molecules and stress response pathways [3].

Oxidative stress occurs when there is an imbalance between the production of reactive oxygen species (ROS) and antioxidant defenses, leading to ROS levels that exceed the antioxidant capacity required to maintain the intracellular redox environment in a reduced state, ultimately resulting in cellular damage. ROS are generated as byproducts of normal cellular metabolism in response to environmental factors. Therefore, the ability of fungi to detect environmental signals, perform robust signal transduction, and trigger accurate responses is fundamental for their survival [4,5].

The systems responsible for removing or detoxifying ROS include superoxide dismutase, catalase, glutathione, and thioredoxins, with glutathione being the primary antioxidant system [5]. Additionally, glutathione is involved in iron metabolism owing to its requirement in Fe-S cluster assembly, sensing and regulation of iron levels, iron trafficking, and biosynthesis of iron cofactors [6,7]

Oxidative stress regulators have already been studied in *Saccharomyces cerevisiae* and other fungal genera of clinical interest, such as *Candida*, *Aspergillus*, and *Cryptococcus* [4]. The prominent roles of three significant modulators have been highlighted: the glycerol high-osmolarity (HOG) pathway, the yeast activation protein 1-like basic-leucine zipper (bZIP) transcription factor, and the response regulator and transcription factor SKN7 [4,5]. The HOG pathway, which is analogous to the p38 and Jun N-terminal kinase pathways in mammals, is highly conserved across the fungal kingdom, regardless of the species niche. This pathway plays a prominent role in adaptation to various stresses in fungal environments, including antifungals, antimicrobial peptides, and osmotic and oxidative stresses, and contributes to virulence [4]. Transcription factors can also orchestrate a robust signaling pathway by either activating or suppressing molecular responses based on the specific biological context to which the fungus is exposed [8,9]. The bZIP family of transcription factors plays a role in stress adaptation in fungi. Among these, the transcription factor activator protein 1 (Ap1) is a major oxidative stress response regulator and is homologous to the mammalian Jun/Fos bZIP transcription factors. Ap1 is activated via oxidation by redox-sensitive glutathione peroxidase 3 in *S. cerevisiae* or thioredoxin peroxidase 3 in *Schizosacchromyces pombe* [4,5]. Oxidized Ap1 accumulates in the nucleus and triggers the expression of oxidative stress-related genes. Additionally, Ap1 interacts with the transcription factor SKN7 to enhance the transcription of oxidative stress-related genes [10]. However, the literature on how dermatophytes respond to oxidative stress is scarce.

The APSES family belonging to transcription factors of the basic helix–loop–helix (bHLH) class, which includes Asm1p, Phd1p, Sok2p, Efg1p, and StuA, is unique to fungi and plays an essential role in regulating fungal growth, virulence, pathogenicity, and metabolism. Regarding transcription factors involved in regulating oxidative stress, previous reports have demonstrated that the APSES transcription factor StuA may play a role in this response [11,12]. However, the mechanism by which StuA regulates the expression of oxidative stress-related genes remains unclear. Additionally, this transcription factor is multifaceted as a critical regulator of metabolism-, adhesion-, and pathogenicity-related genes [11,13,14].

Mining RNA sequencing data from the mutant strain Δ*stuA* of *T. rubrum* during growth in a minimal medium supplemented with keratin [11], we identified that some genes involved in oxidative stress were downregulated in the mutant strain compared with the wild type (WT). Based on these findings, we hypothesized that StuA plays a role in the regulation of oxidative stress-related genes. Therefore, this study aimed to evaluate the transcriptional regulation of oxidative stress pathways, specific genes, and metabolic aspects, focusing on catalase and glutathione detoxification systems by assessing the wild-type (WT) and Δ*stuA* strains during growth in a minimal medium supplemented with keratin (mimicking the primary substrate during an infectious process) and under oxidative stress induced by hydrogen peroxide. Our results demonstrated, for the first time, the importance of StuA in regulating glutathione S-transferases. They deepened our insight into oxidative stress response mechanisms in *T. rubrum* and underscored the pivotal role of transcription factors, such as StuA, in fungal pathogenesis.

## 2. Results

### 2.1. Identification of Genes in T. rubrum Homologous to Oxidative Stress-Related Genes in Other Trichophyton Species

We searched for oxidative stress-related gene homologs in *T. rubrum*, considering that the most involved in oxidative stress pathways are hypothetical proteins. Table 1 shows the homologous oxidative stress-related genes, considering genes with >85% identity.

### 2.2. The Transcription Factor StuA Regulates Genes Related to Oxidative Stress Pathways in T. rubrum

**Figure 1:**
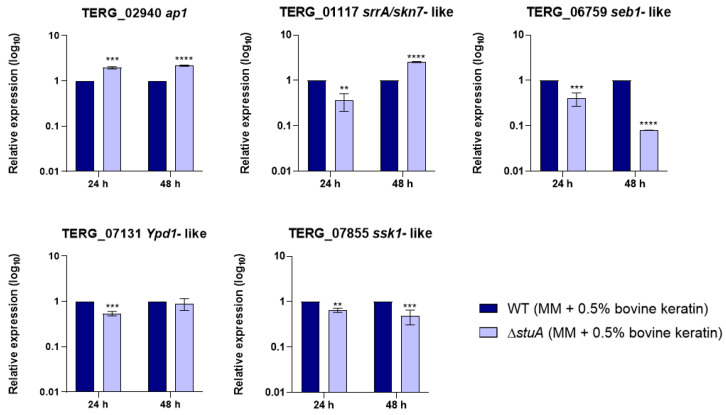
Relative expression analysis of genes involved in oxidative stress pathways during fungal growth in a minimal medium supplemented with keratin (minimal medium (MM) containing 0.5% of bovine keratin) for 24 and 48 h. The WT strain was used as the normalizing condition. Statistical significance was determined using an unpaired Student’s *t*-test with Holm–Sidak correction for multiple comparisons. ** *p* < 0.01, *** *p* < 0.001, and **** *p* < 0.0001.

**Figure 2:**
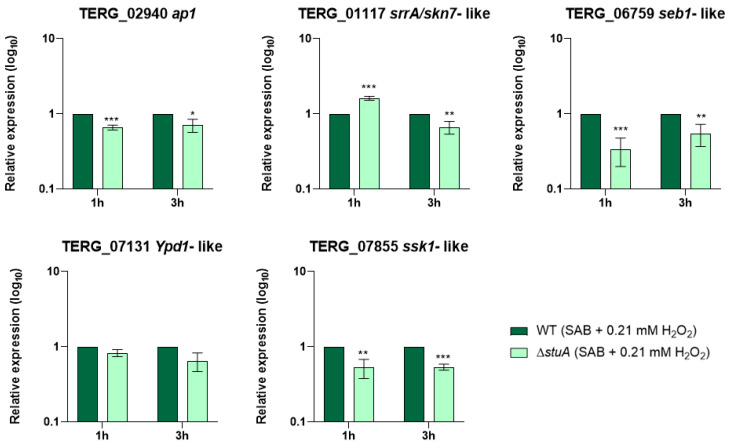
Relative expression analysis of genes involved in oxidative stress pathways during fungal exposure to oxidative stress induction with hydrogen peroxide (Sabouraud medium (SB) with 0.21 mM of hydrogen peroxide (H_2_O_2_)) for 1 and 3 h. The WT strain was used as the normalizing condition. Statistical significance was determined using an unpaired Student’s *t*-test with Holm–Sidak correction for multiple comparisons. * *p* < 0.05, ** *p* < 0.01, and *** *p* < 0.001.

We investigated the influence of StuA on the regulation of the transcriptional response of the central genes involved in oxidative stress pathways during fungal growth in a minimal medium supplemented with keratin (Figure 1) and into oxidative stress induction with fungal exposure to hydrogen peroxide (Figure 2). The MIC value for hydrogen peroxide was established at 0.3 mM for both strains.

Our results showed that the gene encoding the response regulator Ssk1 (TERG_07855) of the HOG pathway was downregulated under both conditions compared with that in the WT strain. The gene *ypd1* (TERG_07131), also involved in the HOG pathway, only reduced transcript levels when Δ*stuA* strain was grown in keratin for 24 h.

Regarding the AP1-SKN7 pathway, we observed that the transcript levels of the gene encoding the transcription factor Ap1 (TERG_02940) were lower in the Δ*stuA* strain than in the WT strain only when fungi were exposed to hydrogen peroxide. The transcriptional response of the gene encoding transcription factor Skn7 (TERG_01117) varied in the mutant strain depending on the duration of fungal culture in keratin or exposure to hydrogen peroxide. The absence of StuA consistently reduced the transcript levels of the gene encoding the Seb1 transcription factor (TERG_06759) under all the conditions evaluated in this study.

### 2.3. Transcript Levels of Glutathione S-Transferase and a Catalase-like Gene Are Reduced in the Mutant Strain

**Figure 3:**
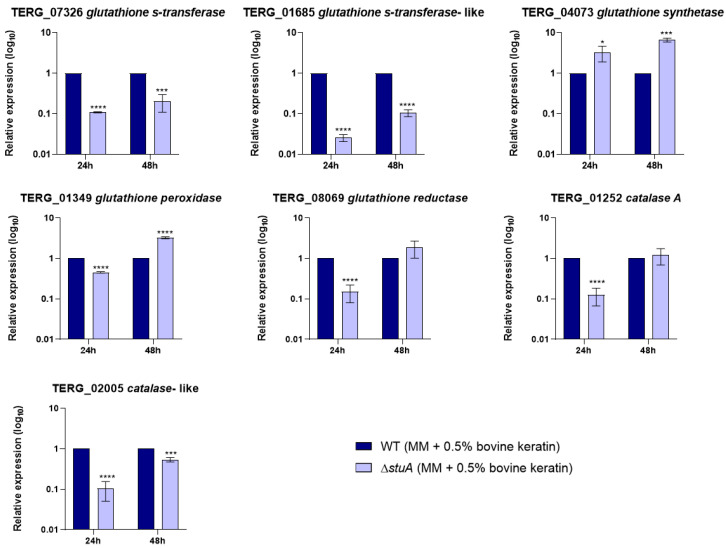
Relative expression analysis of genes encoding the glutathione family and catalases during fungal growth in a minimal medium supplemented with keratin (minimal medium (MM) containing 0.5% bovine keratin) for 24 and 48 h. The WT strain was used as the normalizing condition. Statistical significance was determined using an unpaired Student’s *t*-test with Holm–Sidak correction for multiple comparisons. * *p* < 0.05, *** *p* < 0.001 and **** *p* < 0.0001.

**Figure 4:**
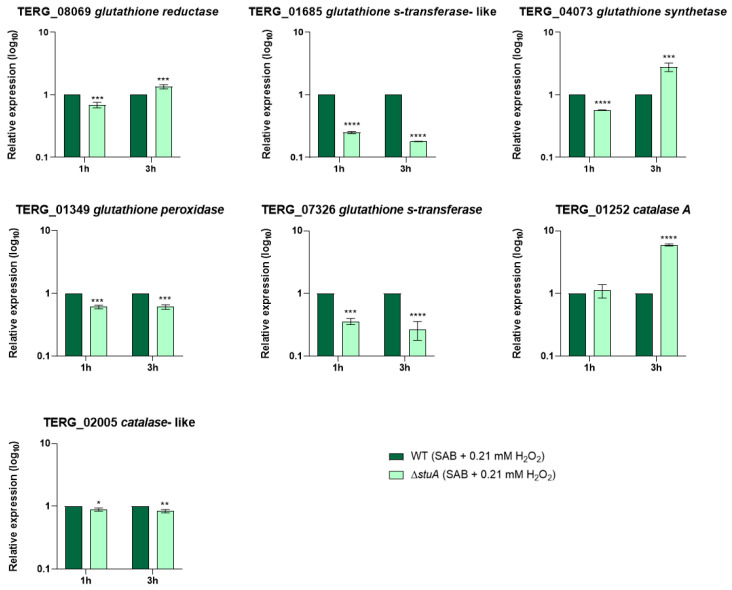
Relative expression analysis of genes encoding the glutathione family and catalases during fungal exposure to oxidative stress induction with hydrogen peroxide (Sabouraud medium (SB) with 0.21 mM hydrogen peroxide (H_2_O_2_)) for 1 and 3 h. The WT strain was used as the normalizing condition. Statistical significance was determined using an unpaired Student’s *t*-test with Holm–Sidak correction for multiple comparisons. * *p* < 0.05, ** *p* < 0.01, *** *p* < 0.001, and **** *p* < 0.0001.

Given that some genes involved in oxidative stress pathways were downregulated in the mutant strain compared with those in the WT strain, we analyzed the transcript levels of genes encoding members of the glutathione family and catalases during fungal growth in a minimal medium supplemented with keratin (Figure 3) and during hydrogen peroxide exposure (Figure 4).

Our results showed that genes encoding glutathione S-transferases (TERG_07326 and TERG_01685) and a catalase-like gene (TERG_02005) were downregulated in the mutant strain compared with those in the WT strain under all conditions evaluated in this study. The glutathione peroxidase-encoding gene (TERG_01349) was downregulated only during hydrogen peroxide exposure. The transcript levels of a glutathione synthetase gene (TERG_04073) were overexpressed in the Δ*stuA* strain compared with the WT strain during fungal growth in a minimal medium supplemented with keratin and after 3 h of hydrogen peroxide exposure. Regarding the glutathione reductase-coding gene (TERG_08069) and the catalase A-coding gene (TERG_01252), the mutant strain maintained expression levels similar to those of the WT strain after 48 h of fungal growth in a minimal medium supplemented with keratin or showed induced expression after 3 h of hydrogen peroxide exposure.

### 2.4. The Transcription Factor StuA as a Consensus-Binding Site for a Gene Encoding a Glutathione S-Transferase in T. rubrum

**Figure 5:**
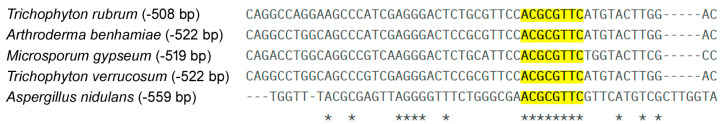
Consensus sequence position for StuA in the promoter region of the gene TERG_07326 of *Trichophyton rubrum*. Multiple sequence alignments of the upstream regions of the Glutathione S-transferase gene across different fungi (dermatophytes and *Aspergillus nidulans*). The consensus-binding sites were determined by identifying DNA sequences upstream (1000 bp) that matched the StuA 5′-[A/T]CGCG[T/A]N[A/C]-3′ DNA-binding consensus. Yellow boxes depict the StuA consensus sequence found in the genic promoters of these fungi. The distance between each consensus sequence and the start codon is shown in the parenthesis. The asterisks (*) indicate positions where all nucleotide sequences have identical bases, showing complete alignment in these specific positions.

We conducted a bioinformatics analysis to determine the existence of a consensus-binding site for the transcription factor StuA in all genes evaluated in this study. Our analysis revealed a consensus-binding site for StuA in TERG_07326, which encodes a glutathione S-transferase (Figure 5). No consensus-binding site for StuA was found in the other evaluated genes.

### 2.5. Evaluation of the Intracellular Catalase Activity of ΔstuA Strain

**Figure 6:**
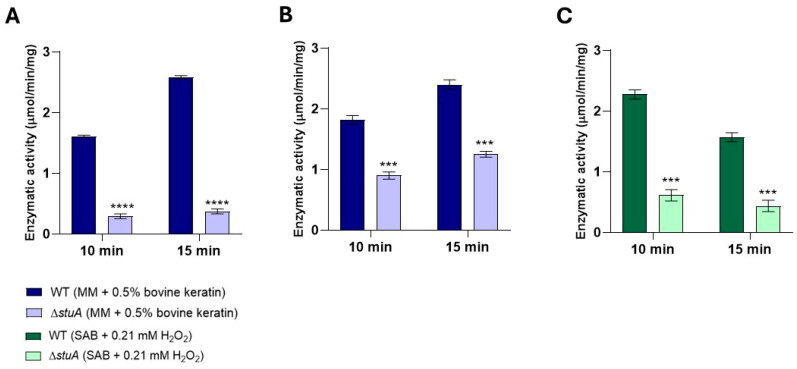
Intracellular catalase enzymatic activity in the Δ*stuA* mutant and WT strains. Catalase activity was measured in protein extracts from fungal growth in a minimal medium supplemented with keratin (minimal medium (MM) containing 0.5% bovine keratin) for 24 h (**A**) and 48 h (**B**) and from fungal exposure to hydrogen peroxide (Sabouraud medium (SB) with 0.21 mM hydrogen peroxide (H_2_O_2_)) for 3 h (**C**). The activity was assessed at 10 and 15 min intervals. The statistical significance was determined using an unpaired Student’s *t*-test with Holm–Sidak correction for multiple comparisons. *** *p* < 0.001 and **** *p* < 0.0001.

To investigate the regulatory role of the transcription factor StuA in the oxidative stress response, we measured the intracellular catalase activity for 10 and 15 min in protein extracts from fungal growth in a minimal medium supplemented with keratin for 24 h (Figure 6A) and 48 h (Figure 6B), and from fungal exposure to hydrogen peroxide for 3 h (Figure 6C). Our results showed reduced catalase activity in the Δ*stuA* mutant strain under all conditions evaluated in this study. Catalase activity was not observed in protein extracts from fungi exposure to hydrogen peroxide for 1 h.

### 2.6. Evaluation of Glutathione S-Transferase Activity of the ΔstuA Mutant During Cultivation in Medium Supplemented with Keratin and Under Hydrogen Peroxide Exposure

**Figure 7:**
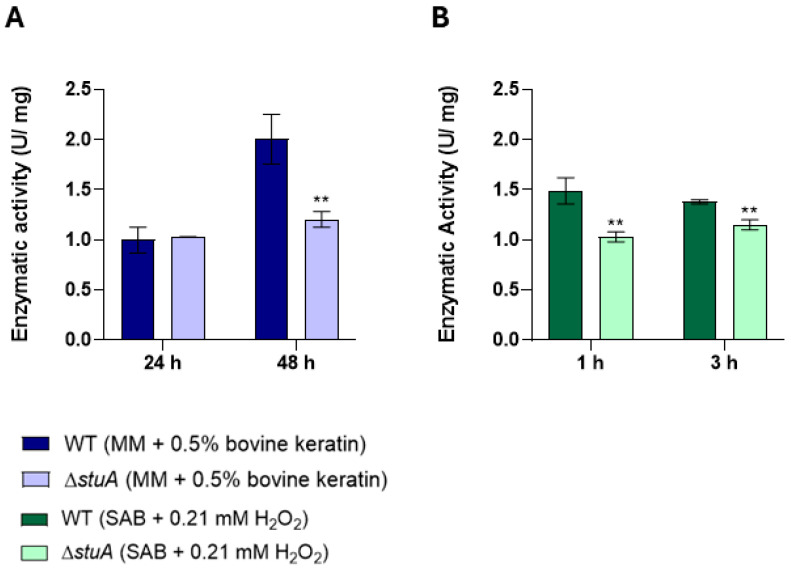
Intracellular glutathione S-transferase activity was measured in the Δ*stuA* mutant and WT strains. The activity was assessed in protein extracts from fungal cultivation in keratin (minimal medium (MM) containing 0.5% bovine keratin) at 24 and 48 h (**A**) and from fungal exposure to hydrogen peroxide (Sabouraud medium (SB) with 0.21 mM hydrogen peroxide (H_2_O_2_)) at 1 and 3 h (**B**). The enzyme activity was measured at 5 min intervals. The statistical significance was determined using an unpaired Student’s *t*-test with Holm–Sidak correction for multiple comparisons. ** *p* < 0.01.

We investigated whether glutathione S-transferase activity was affected by the *stuA* mutation. Our results showed that the activity of glutathione S-transferase was notably impaired in the Δ*stuA* mutant compared with the WT strain during cultivation in keratin (Figure 7A) and under hydrogen peroxide exposure (Figure 7B).

### 2.7. Transcriptional Regulation of Genes Involved in Iron Metabolism When the ΔstuA Strain Is Exposed to Different Culture Conditions

Iron is an essential metal that acts as a cofactor for antioxidant enzymes. However, in excess, it can participate in Fenton reactions, producing highly reactive hydroxyl radicals and exacerbating oxidative stress. In this study, we analyzed two genes involved in the siderochrome iron transport (TERG_03174 and TERG_04949) and a nonribosomal siderophore peptide synthase (TERG_00697) during fungal growth in a minimal medium supplemented with keratin and hydrogen peroxide exposure.

**Figure 8:**
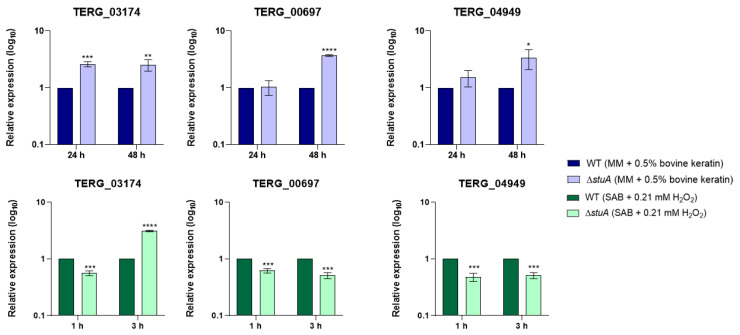
Relative expression analysis of genes encoding siderochrome iron transporters (TERG_03174 and TERG_04949) and nonribosomal siderophore peptide synthase (TERG_00697) during fungal growth in a minimal medium supplemented with keratin (minimal medium (MM) with 0.5% bovine keratin) for 24 and 48 h and during exposure to oxidative stress induced by hydrogen peroxide (Sabouraud medium (SB) with 0.21 mM hydrogen peroxide (H_2_O_2_)) for 1 and 3 h. The WT strain was used as the normalizing condition. Statistical significance was determined using an unpaired Student’s *t*-test with Holm–Sidak correction for multiple comparisons. * *p* < 0.05, ** *p* < 0.01, *** *p* < 0.001, and **** *p* < 0.0001.

Our results reveal the upregulation of these genes in the Δ*stuA* mutant strain, particularly after 48 h of growth in the medium supplemented with keratin. When the mutant strain was exposed to hydrogen peroxide, the transcript levels of these genes decreased compared with those in the WT strain, except for TERG_03174, which remained upregulated after 3 h of exposure (Figure 8).

### 2.8. The Intracellular Iron Content in the ΔstuA Strain During Exposure to Hydrogen Peroxide and Growth in Minimal Media Supplemented with Keratin

**Figure 9:**
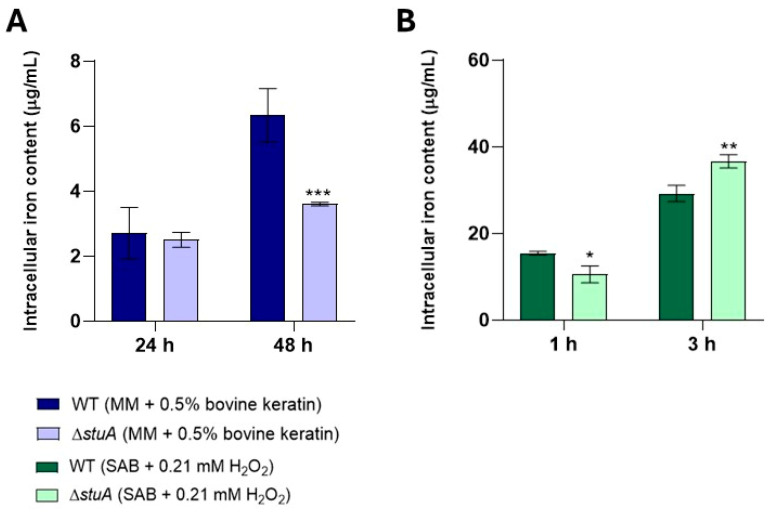
Intracellular iron levels in the Δ*stuA* mutant and WT strains. Intracellular iron levels were measured in protein extracts from fungal growth in a minimal medium supplemented with keratin (minimal medium (MM) containing 0.5% bovine keratin) for 24 and 48 h (**A**) and from fungal exposure to hydrogen peroxide (Sabouraud medium (SB) with 0.21 mM hydrogen peroxide (H_2_O_2_)) for 1 and 3 h (**B**). Statistical significance was determined using an unpaired Student’s *t*-test with Holm–Sidak correction for multiple comparisons. * *p* < 0.05, ** *p* < 0.01, and *** *p* < 0.001.

We also investigated the intracellular iron content in both the WT and mutant strains during cultivation in the presence of keratin and under oxidative stress. Our results revealed higher intracellular iron content in the WT strain after 48 h of growth in a minimal medium supplemented with keratin. However, when exposed to oxidative stress with hydrogen peroxide, the Δ*stuA* mutant strain exhibited increased intracellular iron levels after 3 h (Figure 9).

## 3. Discussion

Regulation of the oxidative stress response is crucial for pathogen survival and thriving in the host. Transcription factors play an essential role in modulating the expression of genes involved in detoxifying ROS and managing oxidative stress pathways, thereby maintaining cellular redox homeostasis.

In this study, we investigated the role of the transcription factor StuA in the regulation of oxidative stress response pathways in *T. rubrum*. Our findings revealed, for the first time, that StuA significantly influences the expression of genes involved in oxidative stress and other related transcription factors. Several genes, including those involved in oxidative stress pathways, are identified in *T. rubrum* as encoding hypothetical proteins. Therefore, we conducted an orthological analysis to identify potential orthologs of these genes in *T. rubrum* based on other dermatophytes.

The absence of StuA substantially impaired the expression of TERG_07855, a gene that encodes the response regulator (*ssk1*), which is crucial for activating Hog1. The Hog1 pathway, one of the most evolutionarily conserved pathways in eukaryotes, forms the backbone of stress signaling via a two-component system-dependent mechanism [15]. Although not present in mammals, the eukaryotic two-component system involves a multistep histidine–aspartate phosphorylation mechanism consisting of a hybrid histidine kinase and a histidine-containing phosphotransfer protein, such as TERG_07131, which encodes the *Ypd1* gene. Histidine-containing phosphotransfer mediates histidine kinase and the response regulator *ssk1*, leading to a cascade of mitogen-activated protein kinase phosphorylation and subsequent Hog1 activation [15,16]. Our results suggest that even if *Ypd1* expression is not significantly affected by StuA deletion during fungal growth in a medium supplemented with keratin or hydrogen peroxide exposure, the cascade of mitogen-activated protein kinase phosphorylation leading to Hog1 activation may be compromised because of the lower transcript levels of *ssk1* in the mutant strain than in the WT strain under all conditions evaluated. Additionally, reduced levels of *hog1* transcripts in the *T. rubrum* Δ*stuA* mutant during fungal cultivation in a medium supplemented with keratin [14] suggest that StuA may play a critical role in modulating the oxidative stress response by influencing key components of this pathway. In addition, Hog1—referred to as HogA in *Eurotiomycetes*—regulates stress-induced transcription and is also involved in sensing sulfur starvation [17]. It is worth noting that keratin also serves as a sulfur source, which could be relevant in this context.

Our research reveals a complex interaction between transcription factors in response to environmental stress. We observed that the transcription levels of the *ap1* gene (TERG_02940) vary according to the growth medium used in this study. However, because the composition of the two growth conditions (minimal medium containing keratin and Sabouraud medium) differs, it is not possible to compare transcriptional regulation directly. Ap1 can also interact with other transcription factors, such as Skn7, which is involved in the antioxidant response. Approximately half of the genes regulated by Ap1 require interaction with Skn7, whereas the other half are independent of Skn7 [18]. The exact mechanism of Skn7 activation via phosphorylation remains unclear. However, once activated, Skn7 cooperates with oxidized Ap1 to regulate oxidative stress-related genes [4]. In the present study, we showed that the skn7 coding gene (TERG_01117) did not show a consistent pattern of regulation by StuA, fluctuating between upregulation and downregulation across different conditions and time points.

Analysis of a Δ*ap1* strain of *T. rubrum* indicated that Ap1 acts as a negative regulator of specific virulence attributes. However, deletion of *ap1* did not affect susceptibility to oxidative stress induced by hydrogen peroxide [19]. To the best of our knowledge, there is no evidence of an interaction between Ap1 and StuA.

Additionally, the gene encoding the C2H2 transcription factor Seb-1 (TERG_06759) was consistently downregulated in the Δ*stuA* strain compared with the WT strain, suggesting that StuA positively regulates this transcription factor. Although not specifically a part of the oxidative stress response pathway, Seb-1 binds to the Stress Response Element under heat stress. In *Neurospora crassa*, a Δ*seb1* mutant strain was more sensitive to oxidative stress during hydrogen peroxide exposure [20], and Seb-1 is also involved in oxidative stress sensitivity in *Valsa mali* [21]. However, there is no evidence that StuA directly regulates or cooperates with Seb1 or other transcription factors. Additionally, no consensus-binding site for StuA was identified in any of the genes involved in the oxidative stress pathways evaluated in this study, suggesting that this regulation might be indirect or mediated through other transcription factors not addressed here.

Considering the obtained results regarding genes involved in oxidative stress pathways, we hypothesize that the Δ*stuA* mutant strain may be more susceptible to oxidative stress effects during hydrogen peroxide exposure owing to the reduced transcript levels of essential genes in the main AP1-SKN7 and HOG1 oxidative stress response pathways.

Regarding antioxidant enzyme-coding genes, we focused on evaluating the transcript levels of the glutathione family and catalases. Our results suggest that StuA can indirectly regulate the transcriptional response of TERG_02005, a catalase-like gene, as the transcript levels were lower in the mutant strain than in the WT under all conditions evaluated. In contrast, the catalase A-coding gene (TERG_01252) did not show a consistent regulation pattern, alternating between down and upregulation depending on time and culture conditions. The regulatory mechanisms of specific gene transcription factors are complex and involve several factors. Similarly, previous RNA sequencing data of the Δ*stuA* strain during growth in a minimal medium supplemented with keratin showed that catalase genes in *T. rubrum* were downregulated [11].

Additionally, our analysis of intracellular catalase enzymatic activity showed reduced activity in the mutant strain in protein extracts during fungal growth in a media supplemented with keratin for 24 and 48 h and in the protein extract after fungal exposure to hydrogen peroxide for 3 h. These findings further confirm the reduced levels of transcription and enzymatic activity of catalases in mutants lacking the transcription factor StuA, which has significant implications for our understanding of the critical role of this transcription factor in regulating catalase activity and managing oxidative stress responses [12,14,22].

Our findings also indicate that the absence of StuA affects the transcriptional regulation of multiple glutathione-related genes. Specifically, StuA appears to act as both a positive and negative regulator of transcript levels for genes encoding glutathione synthetase (TERG_04073), which is responsible for glutathione synthesis, and glutathione reductase, which maintains reduced glutathione levels in cells for cellular homeostasis, depending on time and culture conditions. The absence of StuA diminished the transcription of the glutathione peroxidase gene (TERG_01349) in the presence of hydrogen peroxide, suggesting that the ability to reduce peroxide in cells could be compromised in the mutant strain.

Our study showed that the absence of StuA significantly affected the transcriptional regulation of glutathione S-transferase coding genes, leading to a downregulation of TERG_07326 and TERG_01685 in the Δ*stuA* strain compared with the WT strain across all conditions evaluated in this study. Our study showed that the absence of StuA significantly affected the transcriptional regulation of glutathione S-transferase coding genes. This led to a downregulation of both TERG_07326 and TERG_01685 in the Δ*stuA* strain compared with the WT strain across all conditions evaluated in this study.

Furthermore, we identified a consensus StuA-binding site in TERG_07326, suggesting direct regulation by this transcription factor. Analysis of the enzymatic activity of this class of enzymes also revealed diminished activity in the mutant strain.

Glutathione S-transferases (GSTs) are multifunctional enzymes primarily involved in detoxification. They catalyze the conjugation of glutathione to various toxic compounds, facilitating their excretion from the cells. Additionally, GSTs are involved in cell signaling, regulation of apoptosis, and resistance to chemotherapeutic drugs [23]. Accordingly, mutants with deletions in the GST gene of various fungi show increased sensitivity to xenobiotics and different stressors [7]. For instance, in *S. pombe*, *gst1*Δ, *gst2*Δ, and *gst3*Δ mutants were sensitive to the antifungal fluconazole, indicating the role of GSTs in mediating drug resistance [7]. In *S. cerevisiae*, *gtt1*Δ *gtt2*Δ, and *gtt1*Δ*gtt2*Δ mutants displayed increased sensitivity to heat shock or growth defects at high temperatures. In *Aspergillus nidulans*, the *gstA*Δ mutant was sensitive to heavy metals [7,24]. To the best of our knowledge, no previous studies have investigated the role of StuA in regulating glutathione family genes. However, it is plausible to hypothesize that a deficiency in the conjugation and excretion of toxic compounds in the glutathione-mediated detoxification system may occur in the mutant strain, which could increase its sensitivity to toxic compounds and oxidative stress.

Deficiency of the glutathione-mediated detoxification system leads to an imbalance in iron metabolism. Abnormal iron metabolism causes iron overload, leading to the Fenton reaction and increasing the generation of ROS [6,25,26]. Iron acquisition is essential during the infectious process of dermatophytosis because this metal is vital for many cellular functions, including respiration and energy production. Iron plays a crucial role in the survival and virulence of pathogens during dermatophyte infections. Dermatophytes such as *T. rubrum* need to acquire iron from the environment to sustain their growth and pathogenicity, mainly when infecting keratinized tissues such as the skin and nails [27,28].

Our results showed that genes encoding siderochrome iron transporters and a nonribosomal siderophore peptide synthase, which are essential for iron acquisition, transport, and metabolism, were upregulated in the mutant strain during fungal growth in a minimal medium supplemented with keratin compared with the WT strain. The WT strain presented higher intracellular iron content under the same growth conditions. It is possible to hypothesize that during growth in minimal media supplemented with keratin, the absence of StuA may lead to the upregulation of genes involved in iron metabolism as a compensatory strategy to enhance the uptake of this essential microelement and mitigate the harmful effects of the mutation. However, despite this upregulation, there was no accumulation of intracellular iron, suggesting that iron transport and distribution within mutant cells may also be compromised.

During exposure to hydrogen peroxide, we observed that most genes involved in iron metabolism were downregulated in the mutant strain compared with those in the WT strain. However, the intracellular iron levels of the mutant strain increased from 3 h onwards, which may indicate that despite the lower transcript levels of iron metabolism genes in the mutant strain, there was an increase in intracellular iron as a strategy to enhance the efficiency of its antioxidants, as iron can act as a cofactor for catalases [29], which was shown to be impaired in our results. Alternatively, it could also indicate increased cellular intoxication due to a deficiency in GSTs or other components of the glutathione system, potentially leading to significant cellular toxicity.

## 4. Materials and Methods

### 4.1. Search for Oxidative Stress-Related Gene Homologs in T. rubrum CBS118892

The orthologs of the stress response transcription factor SKN7, C2H2 transcription factor SEB1, phosphotransfer protein Ypd1, response regulator SSK1, glutathione S-transferase, and catalase homolog proteins in *T. rubrum* CBS 118892 were searched using *T. benhamiae*, *T. equinum*, *T. verrucosum*, and *T. tonsurans*, respectively, as queries (ARB_07479, ARB_00366, TEQG_03843, TRV_02413, ARB_02229, and TESG_07337), available at Ensembl Fungi https://fungi.ensembl.org/ (accessed on 9 August 2024). We performed multiple sequence alignments using the Standard Protein BLAST (Basic Local Alignment Search Tool, National Center for Biotechnology Information, USA; https://www.ncbi.nlm.nih.gov/, accessed on 13 August 2024), using non-redundant protein sequences as a database.

### 4.2. Fungal Strains and Culture Conditions

The *T. rubrum* strain CBS118892 (Westerdijk Biodiversity Institute, Utrecht, The Netherlands) was used as a reference (WT), as well as the previously constructed null mutant strain Δ*stuA* [13]. The strains were grown on solid malt extract agar medium (2% glucose, 2% malt extract, 0.1% peptone, and 2% agar, pH 5.7) at 28 °C for 20 days. A conidial suspension was then prepared by flooding the plates with a 0.9% sterile NaCl solution and filtering the suspension through the fiberglass to remove hyphal fragments. Conidial concentration was estimated using a Neubauer chamber. Approximately 1 × 10^6^ conidia of each strain were inoculated into 100 mL of Sabouraud dextrose broth and incubated at 28 °C for 96 h in an orbital shaker with agitation (120 rpm).

For experiments using keratin-containing medium, the resulting mycelia were transferred to 100 mL of minimal medium [30] containing 70 mM sodium nitrate (Sigma-Aldrich, St. Louis, MO, USA) and 0.5% bovine keratin (*m*/*v*). The cultures were incubated for 24 and 48 h at 28 °C with constant agitation (120 rpm). The biological material from three independent replicates was filtered at each time point and stored at −80 °C until RNA extraction.

### 4.3. Oxidative Stress Induction with Hydrogen Peroxide

For experiments involving fungal growth in a medium containing hydrogen peroxide, we first performed a minimum inhibitory concentration (MIC) assay according to the M38-A reference method recommended by the Clinical and Laboratory Standards Institute, with the following modification: 100 μL of the conidial suspension, amounting to 6 × 10^4^ conidia/mL of WT and mutant strains, was added to each well in a 96-well plate. The final conidial concentration was adjusted to approximately 3 × 10^4^ conidia per well in the Sabouraud medium. The MIC was performed with serial dilutions of hydrogen peroxide, ranging between 1.2 and 0.018 mM.

Next, conidial suspensions of both strains were prepared as described in Section 4.2. The resulting mycelia were transferred to 100 mL of Sabouraud medium containing 0.21 mM hydrogen peroxide (70% of the MIC value for both strains). The cultures were incubated for 1 and 3 h at 28 °C with constant agitation (120 rpm). Sabouraud medium without hydrogen peroxide was used as the control.

Subsequently, biological material from three independent replicates of Sabouraud cultures with hydrogen peroxide was filtered under vacuum using filter paper to obtain dry mycelium at each time point and stored at −80 °C until RNA extraction was performed.

### 4.4. RNA Extraction and cDNA Synthesis

According to the manufacturer’s instructions, total RNA was extracted using the Illustra RNAspin Mini Isolation Kit (GE Healthcare, Chicago, IL, USA). RNA concentration and purity were assessed using a NanoDrop ND-1000 spectrophotometer (Thermo Fisher Scientific, Waltham, MA, USA). Total RNA was treated with DNase I (Thermo Fisher Scientific, Waltham, MA, USA) to prevent genomic DNA contamination. Subsequently, cDNA synthesis was performed using the Platus Transcriber RNase H-cDNA First Strand Kit (Sinapse Inc., Miami, FL, USA) following the manufacturer’s instructions.

To assess the quality of the cDNA, we conducted a polymerase chain reaction using oligonucleotides to amplify a region of the constitutive β-tubulin gene, followed by analysis using agarose gel electrophoresis. cDNA was diluted to 70 ng/μL for reverse transcription–quantitative polymerase chain reaction.

### 4.5. Reverse Transcription–Quantitative Polymerase Chain Reaction Analyses

A QuantStudio 3 Real-Time PCR System (Applied Biosystems, Waltham, MA, USA) was used with the primers listed in Supplementary Table S1 for transcript quantification. The concentration of each primer was standardized to achieve reaction efficiencies between 90% and 110%. The reactions were prepared using Power SYBR™ Green PCR Master Mix (Applied Biosystems, Waltham, MA, USA) with ROX dye as a fluorescent normalizer [31]. The 2−ΔΔCt method was used for relative expression analysis [32], with the *T. rubrum* genes *gapdh* and *rpb2* serving as robust endogenous controls [33]. The results are presented as the mean relative expression values from three independent replicates with standard deviations. The induction of oxidative stress by hydrogen peroxide was evaluated in the Sabouraud medium, with the condition without hydrogen peroxide as a control. For relative expression analysis, the WT strain grown in Sabouraud medium with hydrogen peroxide was used as the normalizer condition.

### 4.6. Metabolic Assays

The crude extract of WT and Δ*stuA* strains after growth in minimal media supplemented with keratin or upon hydrogen peroxide exposure was used for metabolic assays. Approximately 0.75 g of macerated mycelium was mixed with 500 μL of a Tris-HCl buffer (50 mM Tris-HCl, 2 mM MgCl2, 2 mM dithiothreitol, pH 8.0). The samples were vortexed and centrifuged for 30 min at 1.268× *g* at 4 °C. The supernatant (protein extract) was collected and stored at −80 °C until catalase and glutathione S-transferase activity assay were performed. Proteins were quantified using the Bradford reagent (Sigma-Aldrich, St. Louis, MO, USA), and concentrations were determined using a standard curve of serial dilutions of Bovine Serum Albumin (BSA) (Sigma-Aldrich, St. Louis, MO, USA).

#### 4.6.1. Catalase Activity Assay

For the catalase activity assay, a hydrogen peroxide solution (2 mL at a final concentration of 10 mM) prepared in 50 mM phosphate buffer (pH 7.0) was used as a substrate for the reaction, followed by the addition of 100 μg of protein extract diluted in 50 μL of the appropriate buffer, as described previously [14]. The decomposition of hydrogen peroxide was monitored over time by measuring the absorbance at 240 nm for 10 and 15 min at room temperature. To infer the enzymatic activity of catalase in each protein extract, the amount of hydrogen peroxide consumed per minute per milligram of protein was measured. The phosphate buffer with crude extract was used as a negative control. Values are presented as means followed by the standard deviation of three independent biological replicates.

#### 4.6.2. Intracellular Iron Assay

For the intracellular iron assay, we used a Serico Iron Kit (Labtest Diagnóstica, Lagoa Santa, MG, Brazil) according to the manufacturer’s instructions. Absorbance readings were taken at 550 nm using a Multiskan™ Microplate Photometer (ThermoFisher Scientific, Waltham, MA, USA), and the results are presented as means followed by the standard deviation of three independent biological replicates.

#### 4.6.3. Identification of Consensus-Binding Sites for StuA

The 5′ upstream regions (1 kb) of all genes evaluated in this study were extracted for analysis. The presence of StuA-response elements (StRE) binding sites, characterized by the consensus sequence 5′-[A/T]CGCG[T/A]N[A/C]-3′, was identified using an ad hoc Perl script based on sequence similarity. Transcription factors of the APSES family (Asm1p, Phd1p, Sok2p, Efg1p, and StuA) contain a basic helix–loop–helix (bHLH) DNA-binding domain, which enables binding to specific stress response elements (STREs) with the consensus sequence [A/T]CGCG[T/A]N[A/C] [34,35].

#### 4.6.4. GST Enzymatic Activity Assay

GST-specific enzymatic activity was quantified using a GST Activity Assay Kit (Elabscience, Houston, TX, USA) according to the manufacturer’s instructions. First, 100 μg of protein extract was mixed with the appropriate buffer and substrate solution. The GST activity was calculated as U/mg by measuring the rate of increase in absorbance at 340 nm. The results are presented as the mean ± standard deviation of three independent biological replicates. One unit of GST activity was defined as the amount of enzyme in 1 mL of the sample that catalyzed the conjugation of 1 μmol of 1-chloro-2,4-dinitrobenzene with reduced glutathione at 37 °C per minute.

### 4.7. Statistical Analysis

An unpaired *t*-test was used to statistically analyze transcript quantification and enzymatic assay results. Statistical significance was determined using the Holm–Sidak method, with *p* < 0.05. Significance is represented in the graphs as * *p* < 0.05, ** *p* < 0.01, *** *p* < 0.001, and **** *p* < 0.0001. GraphPad Prism software v.6 (GraphPad Software, San Diego, CA, USA) [36] was used for statistical analysis and graph design.

## 5. Conclusions

Our results showed that the transcription factor StuA is crucial for regulating genes involved in oxidative stress response pathways and iron homeostasis in *T. rubrum*. The absence of StuA impairs the activation of Hog1 pathway genes, negatively affecting the expression of oxidative stress-related genes, such as *ap1* and *seb-1,* while altering intracellular iron levels, catalase, and glutathione S-transferase activity. These findings highlight the role of StuA in the fungus’s adaptation and survival under stress, thereby enhancing our knowledge of the mechanisms underlying the pathogenicity of *T. rubrum* during infection.

However, to gain a more comprehensive understanding of StuA’s regulatory role in oxidative stress-related genes, future experiments must delve into its regulation under various oxidative stress inducers. This approach will provide a more nuanced understanding of how StuA operates under different stress conditions and the temporal dynamics of gene expression regulation during stress adaptation and infection.

## Figures and Tables

**Table 1:** Oxidative stress-related genes with homologs in various *Trichophyton* species.

Gene ID	Gene Product Name	Homologous
TERG_01117	Stress response transcription factor SrrA/Skn7	ARB_07479 (*Trichophyton benhamiae*)
TERG_06759	C2H2 transcription factor (Seb1)	ARB_00366 (*Trichophyton benhamiae*)
TERG_07131	Phosphotransmitter protein Ypd1	TEQG_03843 (*Trichophyton equinum*)
TERG_07855	Response regulator ssk1	TRV_02413 (*Trichophyton verrucosum*)
TERG_01685	Glutathione S-transferase	ARB_02229 (*Trichophyton benhamiae)*
TERG_02005	Catalase	TESG_07337 (*Trichophyton tonsurans*)

TERG (*T. rubrum* gene identification), ARB (*T. benhamiae* gene identification), TEQG (*T. equinum* gene identification), TRV (*T. verrucosum* gene identification), TESG (*T. tonsurans* gene identification).

## Data Availability

The data presented in this study are available in this article and the accompanying Supplementary Data.

## Supplementary Materials

The following supporting information can be downloaded at https://www.mdpi.com/article/10.3390/ijms252312959/s1.
