# Supplementary material for: The Transcription Factor StuA Regulates Oxidative Stress-Responsive Genes in Trichophyton rubrum
Source: Int J Mol Sci. 2024 Dec 2;25(23):12959. doi: 10.3390/ijms252312959 (PMC11641030; doi:10.3390/ijms252312959)
Supplement: Supplementary file 1 [file ijms-25-12959-s001.zip › ijms-3302962-supplementary.pdf]

Table S1. Primer sequences used for RT-qPCR analysis

Gene ID	Gene Product Name	Primer sequence (5′-3′)	Amplicon
TERG_02940	bZIP ap1 transcription factor	F: GCCACTTTGATCCAGTTCTG<br>R: TCAGGTAGAGGGAAAGCATC	103 bp
TERG_01117	Stress response transcription factor SrrA/Skn7	F: TCTACTCTTTCTCCTGCGTC<br>R: CCTGTATCTTGTTCACCGC	117 bp
TERG_06759	C2H2 transcription factor (Seb1)	F: CGAACTGCTCAGAGTAATGTG<br>R: GGTGCTTTGTGGATTGGTC	120 bp
TERG_07131	Phosphotransmitter protein Ypd1	F: TGTCCCAACTAGGTCACTTC<br>R: TGCCAGACTCGTCCTTTT	114 bp
TERG_07855	Response regulator ssk1	F: GGATGGCGGTTTGTCAAC<br>R: TGGAGGCTACAGGTAATCTTC	95 bp
TERG_07326	Glutathione S- transferase	F: GAGCACAAGAAGAAGCTGGA<br>R: AGCCAAGAAGGGAAGGTAATAG	127 bp
TERG_01685	Glutathione S- transferase	F: GACTTCAGACAGTGCAAGGA<br>R: ACCAGATTCCCAGAGGTAGA	105 bp
TERG_04073	Glutathione synthetase	F: CGTTCACGACGCATATTCAATG<br>R: GACCGATTCTCGCCTTACTTATC	99 bp
TERG_01349	Glutathione peroxidase	F: TCAACTACGGCGTCACTTTC<br>R: AGACCAGGCATCTCCTTCT	100 bp
TERG_08069	Glutathione reductase	F: CCCGTTCTACTCCTCCTCTATT<br>R: TAGTCGCCAGTGTTCTGTTG	101 bp
TERG_02005	Catalase	F: CAGGAGTCCGCTCATCAAAT<br>R: CTTGTATGTGTGGCCAGAGTAG	96 bp
TERG_01252	Catalase A	F: ATTGGGATCTCGTGGGAAAC<br>R: GGCCTTGAGGGACTTCATTAT	114 bp
TERG_03174	Siderochrome iron transporter	F: TGTCGACGCAATGGAAG<br>R: AGGTTGATTCCCAGTAAAGCC	85 bp
TERG_00697	Nonribosomal siderophore peptide synthase	F: GACTGTGGTTCCCACTGAAA<br>R: TCGGTTGGCTGCTGTAAAT	105 bp
TERG_04949	Siderophore iron transporter mirC	F: CCATTCTGGGAGACATCCAAA<br>R: GAAGAAAGCCAATGCACATCC	102 bp
rpb2	DNA-directed RNA polymerase II core subunit RPB2	F: TGCAGGAGGTTTGATGAAGA<br>R: GCTGGGAGGTACTGTTTGATCAA	59 bp
gapdh	Glyceraldehyde-3-phosphate dehydrogenase	F: GCGTGACCCAGCGATGTAGT<br>R: CGGTGGACTCGACGATGTAGT	62 bp
